# “I Don’t Do Anything; I’m Just Being Taken Care Of”: Experiences of Patients and Their Caregivers Transitioning Back into the Community Following Traumatic Injury in Northern Tanzania

**DOI:** 10.3390/traumacare2020028

**Published:** 2022-06-03

**Authors:** Anna Tupetz, Loren K. Barcenas, Julia E. Isaacson, Joao Ricardo Nickenig Vissoci, Victoria Gerald, Julius Raymond Kingazi, Irene Mushi, Timothy Antipas Peter, Catherine A. Staton, Blandina T. Mmbaga, Janet Prvu Bettger

**Affiliations:** 1 Division of Emergency Medicine, Department of Surgery, Duke University School of Medicine, Durham, NC 27710, USA; 2 School of Medicine, Duke University, Durham, NC 27710, USA; 3 Duke Global Health Institute, Duke University, Durham, NC 27710, USA; 4 Kilimanjaro Christian Medical Center, Moshi, Tanzania; 5 Paediatric and Child Health Department, Kilimanjaro Christian Medical University College, Moshi, Tanzania; 6 Clinical Trial Department, Kilimanjaro Clinical Research Institute, Moshi, Tanzania; 7 Duke-Margolis Center for Health Policy, Duke University School of Medicine, Durham, NC 27710, USA; 8 Department of Orthopaedic Surgery, Duke University School of Medicine, Durham, NC 27710, USA

**Keywords:** transitions of care, Tanzania, injury, disability, biopsychosocial, trauma, function

## Abstract

After discharge from the hospital for traumatic injury, patients and their caregivers face a period of increased vulnerability. This adjustment phase is poorly characterized, especially in low- and middle-income countries. We explored the experiences of patients and their caregivers in Northern Tanzania after hospitalization for a traumatic injury. Patients who received care for traumatic injury at the Kilimanjaro Christian Medical Center and their caregivers were selected as part of a convenience sample from January 2019 to December 2019. Analysts developed a codebook; content and analytic memos were subsequently created. We then applied the biopsychosocial model to further characterize our findings. Participants included 26 patients and 11 caregivers. Patients were mostly middle-aged (mean age 37.7) males (80.8%), residing in urban settings (57.7%), injured in road traffic accidents (65.4%), and who required surgery (69.2%). Most caregivers were female. Seven major themes arose: pain, decreased physical functioning, poor emotional health, lack of support, challenges with daily activities, financial strain, and obstacles to accessing healthcare. This study describes some of the difficulties transitioning back into the community after hospitalization for traumatic injury. Our work demonstrates the importance of mixed methods approaches in characterizing and addressing transitions of care challenges.

## 1. Introduction

Each year, over one billion people worldwide suffer traumatic injuries and approximately 5.8 million people die due to their injuries [1,2]. The burden is not distributed equally; rather, more than 90% of injury-related deaths occur in low- and middle-income countries (LMICs) [3]. LMICs, defined by the World Bank in 2022 as countries with a gross national income per capita between $1046 and $4095, describe multiple African nations including Tanzania [4]. Tanzania faces a high injury burden with around 10% of patients who present to emergency departments reporting trauma-related complaints [5,6]. The economic burden of trauma is considerable. Road traffic crashes alone, the mechanism of injury accounting for 23% of reported traumas, cost LMICs $65 billion annually [3,7]. Further, nearly 50% percent of injuries occur in young persons aged 15 to 44, thus disrupting what are likely to be those individuals’ most economically productive years [8]. A prospective study conducted at the Kilimanjaro Christian Medical Center, a large tertiary referral hospital in the Moshi region and the setting of this work, reported a significant loss of employment and/or lower wages in patients who experienced non-fatal musculoskeletal injuries [9].

After discharge from the hospital for traumatic injury, nearly all patients will face a period of adjustment when they return home. For some, this may include living with pain or a new disability that impacts mobility and function. Others may face psychosocial or mental health challenges as a result of newfound isolation, financial obligations, and/or dependence on others. In LMICs especially, the cyclical relationship between poverty and disability further complicates these challenges [10,11]. Yet, limited literature, particularly in LMICs, reports on the experiences of trauma patients with a temporary or long-term disability during this transitional period [12,13].

Further, care transition programs that have proven to reduce morbidity, mortality, and disability developed in high-income countries (HICs) have yet to be adapted and evaluated in LMICs [13]. The biopsychosocial model is one important framework to use when considering the development of any patient’s medical state and how it relates to their environment and context, although limited literature exists on its application to transitions of care and injury populations. The biopsychosocial model is based on the idea that to fully understand the patient experience, one must simultaneously acknowledge the biological, psychological, and social aspects of health [14]. The model is well-accepted as a valuable tool, forming the basis of the World Health Organization’s International Classification of Functioning, Disability, and Health [15,16]. Previous work has demonstrated that patients attend more follow-up appointments and experience decreased re-admission rates when their post-discharge care incorporates their biological, psychological, and social needs [17–19].

Determining how to best address patient needs for the transition home after a traumatic injury requires an approach that incorporates the patient, caregivers, healthcare providers, and the community. Understanding the perspectives of key stakeholders during the hospital-to-home transition after traumatic injury is critical in designing systems that best meet patient needs within their specific sociocultural structures. This is especially true in LMICs, such as Tanzania, which face a high burden of injury-related hospitalizations with known challenges in the care process of the affected patient population [6]. Tanzania is a resource-limited country and although economic growth over the past decade has been consistent, more than one-quarter of Tanzanians live in poverty [20]. The country’s healthcare system faces a variety of challenges and much of the population has limited access to healthcare. As of 2019, only 32% of Tanzanians had health insurance although significant efforts are underway to provide universal health coverage [20,21]. Further, a recent study demonstrated that only 32.9% of a sample of East Tanzanian residents had adequate health literacy, an important driver of health outcomes [22].

In this study, we interviewed patients who were hospitalized for traumatic injury and their caregivers in Northern Tanzania to better understand the post-discharge experience. The aim of this study was to explore patient and caregiver experiences through the lens of the biopsychosocial model after hospitalization for traumatic injury.

## 2. Materials and Methods

### 2.1. Study Design

This descriptive qualitative study followed a phenomenological approach with a specific focus on the individual experiences of the interviewed patients and caregivers. This study was approved by the Duke University Medical Center Institutional Review Board, the Kilimanjaro Christian Medical University College Ethics Committee, and the Tanzanian National Institute of Medical Research under the IRB protocol number Pro00086496. We followed the COnsolidated criteria for REporting Qualitative research (COREQ) guidelines.

### 2.2 Research Team and Reflexivity

Three female and two male Tanzanian research assistants, all with bachelor’s or master’s degrees, conducted the interviews after undergoing training in qualitative data collection methods by the research team. Research assistants established rapport with the participants at the beginning of each interview session and explained the study goals. The research assistants were not known to the participants or involved in their clinical care and no personal information about the interviewers was shared with the participants. The investigator team consisted of Tanzanian and US-based clinicians and researchers with doctoral degrees, who have been collaborating on a number of projects for several years. The coding was performed by two US-based research assistants and a co-investigator with qualitative data analysis expertise.

### 2.3. Setting

This study took place in the northwest of Tanzania. Tanzania is a lower-middle income country in East Africa with an estimated population of 59 million [23]. Participants in this study were associated with the Kilimanjaro Christian Medical Center (KCMC), an urban referral hospital in the Kilimanjaro region of northwest Tanzania serving patients from urban and rural populations [24,25]. The hospital is the third largest facility in the country with 630 beds and a regional training center for a variety of healthcare professions. The KCMC offers inpatient as well as outpatient services in multiple specialties, offering follow-up and ongoing medical care to their patients after discharge. The hospital serves more than 60,000 patients yearly and the Emergency Department, from where study participants for this project were initially recruited, sees approximately 2000 injury patients annually [26].

### 2.4 Participants and Recruitment

Eligible participants presenting with traumatic injury were identified through a prospective trauma registry at the KCMC Emergency Department. Eligible participants included adults at least 18 years of age who presented to the Emergency Department with a traumatic injury requiring hospitalization within 24 h of presentation, were medically stable, and resided in Moshi urban or Moshi rural areas. Traumatic injuries were defined as injuries of sudden onset caused by an extrinsic force. Following a convenience sampling approach, we invited eligible patients to participate in in-depth interviews. We approached patients in person while in the hospital. Participation was voluntary. Patients who, after discharge, were incarcerated or admitted to a substance use rehabilitation center and thus did not return to their usual home environment were excluded. The caregivers included were over 18 years of age and lived in close proximity to the patients. Participants signed informed consent forms before beginning their interviews.

### 2.5. Data Collection

All interviews were conducted in person after the patients’ discharge from the hospital from 20 January 2019 to 12 December 2019. Participant interviews took place, on average, 55 days after hospital discharge (min: 6 days; max: 186 days). Patients and caregivers were not in the same room for their respective interviews. Interviews lasted up to 70 min but typically were around 30 min. All interactions were audio-recorded with only the participants and the researchers present. Interview guides were developed in English, translated to Swahili by bilingual research assistants, back-translated, and pilot tested by the research team. All interviews were conducted in Swahili and were transcribed ad verbatim in Swahili to ensure consistency. They were then translated to English by bilingual Tanzanian research assistants.

In July 2019, after six months of data collection and analysis and 20 completed interviews, the research team conducted a preliminary analysis for codebook development. Based on the findings and identified gaps in knowledge, the interview guide was revised and an additional six patient interviews were conducted between October and December 2019. During the revision of the codebook, no substantial changes to the topics covered in the interview guide or to the interview process were made. The meeting solely determined which topic areas required further probing by the interviewers and additional follow-up questions were added where necessary. No repeat data collection was carried out. Field notes were made during each interview and focus group; however, transcripts were not returned to participants for comment or correction.

### 2.6. Data Analysis

Thematic content analysis was the methodological tool underpinning this study, used to determine both major themes and sub-themes. The analysis followed a mix of inductive and deductive coding approaches. The interviews were initially coded inductively. The emergent major themes were then deductively applied to the biopsychosocial model. Coding was performed in a spreadsheet to facilitate collaboration and communication amongst the researchers. Three analysts, with master’s or PhD degrees in scientific research and experienced in qualitative data analysis, and who were not involved in the interviews read the first two transcripts and coded the interviews into themes and categories. A first version of the codebook was developed by the three coders, following a semantic coding approach. The major themes remained consistent throughout the analysis process, though new emergent codes and sub-themes were continuously added and revised throughout the analysis process. In case of disagreements or questions, an agreement was reached through discussions amongst the coders.

Preliminary results and the codebook were discussed with the entire research team and Tanzanian collaborators in July 2019, when a mid-term preliminary analysis was performed. Another revision and discussion of the final codebook occurred with the entire team after data collection terminated in December 2019 to incorporate any changes to the codes and codebook based on the interviews conducted between July and December. The interviewers and our local Tanzanian research team also validated our data for cultural understanding and appropriate interpretations. Participant validation of themes was not performed. The data were then further analyzed and interpreted through the creation of content and analytic memos, which again were shared and discussed within the research team for validation. After completion of data collection and analysis, the biopsychosocial model was applied to the major themes for the organization of further discussion and to inform intervention development strategies. The conclusions drawn from the application of the biopsychosocial model were reviewed and discussed with the research team.

## 3. Results

Participant demographics: Our sample consisted of 26 patients and 11 caregivers. The majority of the interview patient participants identified as male (*n* = 21) and sustained a road traffic injury (*n* = 17), consistent with the characteristics of the larger injury population according to the trauma registry used in this study. Characteristics of the participants are summarized in Table 1.

Major themes from the interviews included experiences and impact of pain; the perceived impact of the injury on health and physical function; emotional and psychological well-being; community, friends, and family support; impact on activities of daily living; finances and income; and access to healthcare including the ability to follow healthcare recommendations. The majority of the major themes also have multiple distinct sub-themes.

Applying the biopsychosocial model to the major themes, biological factors include pain and physical functioning, psychological factors include emotional well-being, and social factors include support and impact on activities of daily living. Finances and access to healthcare are at the intersection of the components of the three-part model and are described as contextual factors. Table 2 provides the full coding overview including sub-themes.

### 3.1. Biological Factors

#### 3.1.1. Experiences and Impact of Pain

Patients and caregivers commonly discussed pain. The level of pain reported ranged from no pain at all to severe pain. For many, pain seriously impacted their daily functioning. Some additionally reported insomnia, mental distress, and limited ability to engage in social interactions due to pain. For some, negative experiences with pain became a source of anger and contributed to mental health and coping challenges. In a few cases, patients chose not to voice concerns about their pain levels in an attempt to remain strong for their caregivers.

INT: Ok, how does pain affect your activities? How does pain affect what activity you are doing?

P567 (M): Since the hand can’t work, so the work I was doing I cannot do.

INT: So, you don’t have any activities that you do right now?

P567 (M): At the moment I have no activities.

#### 3.1.2. Perceived Impact of Injury on Health and Physical Function

Before the injury, most participants reported that they were healthy. After discharge, the most commonly reported health concerns were physical impairments and pain around the injury site. Some voiced that they had experienced dizziness, balance impairments, fevers, cough, and ulcers. A few patients and caregivers reported challenges in responding or listening for extended periods of time and discomfort being in crowded and noisy places. One reported difficulties in communicating. Some patients reportedly experienced memory loss after the injury although the deficits proved temporary.

P111 (M): Yeah, about losing memory, at first there was that problem, but it reached a point where he got it back. But after coming back from the hospital, it was hard with his memory, but after some time at least now we see the memories are coming back.

### 3.2. Psychological Factors

#### 3.2.1. Emotional and Psychological Well-Being

Most patients and caregivers reported at least some level of emotional distress during the transition of care period. However, some were well able to accept the situation in order to tolerate the stress and voiced that they had realistic expectations for the future and were willing to ask for support. Those who did express concerns about their emotional health mentioned uncertainty about the future, impacts on caregivers, and experiencing feelings of loneliness.

##### Uncertainty about the Future

Uncertainty about the expected recovery timeline was a source of mental health issues for many participants.

INT: [ … ]How long do you think that will take? Until you can feel that you are better?

P593 (M): I think not yet, because I listen to the doctors. [ … ]I may wait a long time, I think up to February maybe, I am worried about something like that.

Patients and caregivers were challenged emotionally by the uncertainty in their ability to continue their education, return to work, and support their families. Some were unsure of the next steps in their care plan and if they would develop chronic pain or physical disabilities. Others wondered if they would be able to return to relying on previous modes of transportation. Some worried about how their injury would impact their children’s future. In one case, not knowing what to expect triggered the desire to start drinking.

P240 (M): When I went to see the doctor, he gave me some information that bed rest was part of my healing process, he gave me some advice but still my mind did not agree to it. But in the end, I decided to change myself, but inside my mind I thought I was already incapacitated. I questioned myself, who will take care of my family? Who will take over the work I was doing? Every time I thought about it, I got very stressed. I wanted to start taking alcohol which I was not taking before [the injury].

Patients and their caregivers generally believed that the medical team should be responsible for managing expectations and providing education on the recovery process, thus helping prevent patients from feeling isolated and hopeless upon discharge.

##### Impact on Family Members’ Health

Most mentions of the impacts of injury on the physical and mental health of the patients’ family members were made during interviews with the caregivers. However, some patients did acknowledge the increased emotional burden on their family related to fears of re-injury, caregivers’ perceived responsibility to help prevent further harm, and confusion about the patient’s medical state. A physical manifestation of the caregiver burden was dizziness due to extended periods of lack of sleep.

The family members mostly worried about food insecurity, the pressure of meeting the patient’s needs, coping with the patient’s emotional state, and the necessity of taking on new roles. It was mentioned that it was challenging for the children to see their father in a place of dependence and in need of support. Some voiced to be in a state of shock. There seemed to be a perceived lack of support for the caregivers with one patient mentioning that s/he actively tries to reduce reasons for the caregiver to worry.

##### Impact on Patients’ Mental Health

Generally, patients and caregivers described the emotional impact of the injury as “worrying a lot” or “thinking too much”. The most common emotion described by the patients was a feeling of loneliness and isolation.

P111 (M): Because of my thoughts, pain, loneliness. I want to get out there, but I can’t so that it affects me, I’m lonely, I want to walk but I can’t. For example, I can call a child to bring me drinking water, but the child was washing clothes so that might delay him from coming. I may see him to have despised me although it might not be the case. It has affected my mind a lot.

The families were generally aware that social isolation negatively impacted the emotional well-being of their injured family member but were uncertain how the patient truly felt since the injury. Patients and caregivers had a hard time accepting that family dynamics had shifted. A lack of motivation, stress, frustration and a feeling of insecurity due to the inability to protect oneself was also voiced. Flashbacks to the injury impacted some patients’ ability to sleep. The families and patients acknowledged pain as a source of mental distress and that having found that effective pain management strategies positively influenced their mental well-being. Some patients felt misunderstood and unsupported by their families and communities.

##### Unadjusted Coping/Failure to Ask for Support

In some cases, the feelings of hopelessness were overwhelming. Patients felt like a burden and were uncomfortable asking for help, choosing to wait until their situation was unbearable. Some voiced that there was a limit to how much they could ask for and that they would rather wait until help was offered. One patient mentioned they would feel more comfortable asking for help when they had better financial stability or found a way to pay back in some ways for the support they received.

### 3.3. Social Factors

#### 3.3.1. Community, Friends, and Family Support

##### Definition of Community

The community was defined as relatives, neighbors, friends, church members, work colleagues, and those who help, regardless of how well they have been known by the patients.

P699 (M): Can usually say that my community is the people around me, people close to me, even those who are far from me are my community, because the one who are close to you are not the only one who look after you, those who look after you are many, let me take example from the accident that I got, the moment that I have got an accident, I failed to walk, even if people who don’t know me I believe that for the condition that I had they can help me, therefore sometimes a community can be the people you know and the people you don’t know.

Level of Community Support/Lack of Support at Home Post-Injury from Community Members Most commonly, the patients reported having received little to no community support.

INT: All right, who is helping you and your family in dealing with these problems?

P389 (M): Aah currently there is no one is helping me.

INT: Community, family, church, at work?

P389 (M): There is no [support].

Yet, participants seemed to understand that some community members may be preoccupied with their own lives. One participant acknowledged that you are unlikely to receive high levels of support if you were not an active community member before the injury. The stronger the relationship prior to the injury, the more supportive the community was in assisting with material, financial, and emotional needs. Some reported that neighbors would step in and help when they noticed a need while others mentioned they had to actively ask for help, especially in covering healthcare needs. Generally, the patients did not report any perceived stigma and felt welcomed back into the community.

Community involvement varied in both the level of engagement and the duration of support. Patients reported it was helpful when community members checked in, sent well wishes, and provided emotional support. In addition, types of support included financial assistance and help with food preparation, housework, and daily activities. In some cases, the patients were fully dependent on the community, mentioning that if they did not deliver food for them, they would remain hungry, or if they did not assist them in feeding the chickens, they would not have an income.

Several participants were unaware of any potential community resources that they could access. One patient felt there was a need to educate the communities on how to support patients after returning home from the hospital and how to meet their healthcare needs.

INT: Ok, mmh how have you been accepted or received back into your community due to the new changes in your life?

P313 (M): They feel okay, they feel sick there are things now you see you can do but they see you still can’t do, so you find yourself, it is like they monitor you too much, ‘let us help you, stop doing that,’ but you feel you have the ability to do that. Let’s say it is to that extent but now I don’t know if it helps how the patient feels that there are things he can’t do, until you force him now, you will have to force [the patient] doing things.

##### Injury Leads to Lack of Active Community Engagement

In several cases, the patients reported that their injury, level of mobility, transportation limitations, dependence on caregivers to leave the house with them, and inability to engage in previous activities led to social isolation from the community. They sometimes perceived themselves to be too ill to reintegrate into their previous community relationships. One patient also mentioned that it was not their personality to disclose problems to the community.

##### Level of Support from Religious Community

The religious community was not consistently mentioned as an avenue for support. However, if patients had a pre-established connection to the community, religion was an additional source of hope and encouragement, used as a way to understand and accept hardship and as a tool for family members to console patients. The religious community supported patients and their families through prayers, visits, and help with daily activities. Further, some patients were comforted by the belief that God has a plan and that s/he will recover when and if that is part of the plan. For several patients, it was important to go to church although sometimes access to the religious buildings was difficult due to challenges with mobility. In those cases, the religious community often sought out other ways to interact with the patient and their families.

##### Level of Family Support

In most cases, the family was considered to be the main support system. Families generally tried to cover their needs internally as much as possible without having to seek external community support.

P397 (F): For the things that I need after I got injured, mother is providing everything. Therefore, if I will need anything, mother is always serving; therefore, there is nothing I need.

The distribution of roles among the families varied across family members depending on location and availability, where some were most involved during the hospital stay, whilst others stepped in to help out at home or simply provided emotional support. The nature of familial involvement also depended on which family members were available when the patient was willing to ask for help, and from whom the patient was willing to accept help in certain activities.

Injuries often caused family structures to shift. In some cases, family members stopped working in order to become caregivers. In other cases, family members sought out employment to cover their newfound financial needs. Shifting family dynamics sometimes resulted in inadequate family support systems when the leader of the family, who would usually step in to organize and counsel family members, became the one in need of support.

##### Injury Impacted Family Daily Activities

The children’s daily lives, in some cases, were directly affected if a loss in income resulted in the inability for them to continue school or afford school supplies. Those who were able to continue their education may have experienced a decrease in their performance due to growing caretaking responsibilities at home. The burden on the caregivers, as voiced by the caregivers directly, included the need for them to put their needs and goals aside, leaving them tired and exhausted. Some family members also expressed that their caretaking responsibilities influenced their own work and careers and caused a financial burden due to shifting family dynamics.

INT: Please, can you explain how your normal day has become after the injury, after getting injured when you came to the hospital and got discharged, how your normal day has become.

P359 (F): It is very bad.

INT: Bad in what way?

P359 (F): I cannot do anything anymore, taking care of my children, even cooking for them, I can no longer do that.

##### Expectations for the Support System

Generally, patients did not have high expectations or demands for their support system and often reported discomfort in asking for help. There were very little to no expectations that everyone would be supportive and react positively to their injury. Rather, the patients were understanding of people unable or unwilling to help and if they sometimes reacted harshly or seemingly unfairly.

INT: Ok. What do you expect from the community? The community around you?

P577 (M): Mainly what I expect from the community is just love and unity, that’s it, but I know that is the output of how you invest on them, to me that is how it seemed because I live with people well in the religion, in the working part, with neighbours, even children used to come here, because they missed me, so to me I am very grateful, I feel, I felt different, you know at first when I came from the hospital, because when they came they used to be limited with time, come at this time, come at this time, yeah, it is good for the patient’s health he is supposed to rest but even if I limit their time when I need them I can see them so to me as I sleep I hear them telling stories, the sickness is like going away, half of it, you feel like you are part of the community.

##### Structured/Organized Support Systems

The patients and caregivers were mostly unaware of existing support systems within the community. They voiced the need for paid caretakers or aides in the household but also reported that there was no system in place for finding and paying them. Another challenge for paid caregivers was to find some who were as skilled as family members. Additionally, the patients and families mentioned the need for health professionals within the community to provide communication and recommendations for better recovery while at home.

#### 3.3.2. Impact on Activities of Daily Living

##### Injury Impacting Daily Activities or Ability to do Housework

Most patients’ daily lives were heavily impacted by the injury.

P67 (M): To be honest life has changed a little bit because I don’t do anything; I’m just being taken care of.

Patients’ level of function in daily life activities ranged from full independence to being bedridden when no caregiver was present. The effects of limitations in mobility were wide-ranging, impacting everything from patients’ motivation to continue their education to their ability to move in the house and follow prayer routines to family dynamics.

##### Growing Ability to Perform Daily Activities with Less Support

Recovery or progress was identified by patients and caregivers as increased independence in daily life activities, decreased dependence on caregivers, and a potential return to work. Patients and caregivers identified recovery with increased community reintegration. Patients mentioned that, while progress comes with newfound hope and improved mental well-being, increased independence also comes with new safety concerns.

##### Environmental Changes (House, Assistive Devices, Transportation, etc.)

Only a few participants mentioned the need to make adjustments in the house to increase accessibility and independence. Environmental changes included adjusting transportation means and requiring assistive devices.

### 3.4. Contextual Factors

#### 3.4.1. Finances and Income

##### Injury Negatively Impacted the Ability to Generate Family Income

Almost all participants experienced a negative impact on the ability to generate family income due to the injury suffered. Some patients specifically mentioned that due to his/her injury, generating income working as a farmer or a boda boda driver was no longer possible.

INT: So now, how has it affected you?

P64 (F): I have no income if I fail to feed the chickens, and I have no one to help me. I have no helper or worker.

Several participants reported difficulties in covering the basic needs of the family, including food provision for all family members, and that the generated income by the caregiver was insufficient to cover ongoing costs.

P593 (M): Yes, they are the problems, mostly food, for instance yesterday we didn’t even have anything to eat, you just stay like that, that’s it, this morning I got some tea here from this neighbor, she gave me tea with nothing else, I sat right on the bucket there.

##### Injury-Related Costs Increase the Financial Burden on the Family

Common injury-related costs that the families were unable to cover included transportation to access healthcare, adjustments to improve the accessibility of the home environment, medical treatments including surgery and less expensive alternatives, assistive devices, and medical equipment. Available finances were spent on basic daily needs before covering healthcare needs, leading to delays or even discontinuations of care or further impoverishment of the families requiring external support. Some families were forced into debt in order to cover healthcare costs.

In addition to healthcare costs, indirect spending, such as having to pay for services the patient used to be able to perform themselves, hiring paid caregivers, or altered nutrition to support recovery were mentioned by patients and caregivers.

Participants also mentioned a lack of awareness and transparency of the actual healthcare-related cost, even for those who had health insurance. Not all participants were able to afford health insurance, which led to a discontinuation of care.

P183 (M): There is huge money deficit like when we are required to go to [city] for MRI tests, in fact it has come to a time when we need the help of close relatives because if you look at KCMC, costs are very expensive, from the time he was admitted at KCMC until discharge it was costly, we even used all the savings we had.

##### Found New Ways to Generate Income/Savings to Help Cover Costs

Some families had savings that helped cover ongoing costs and were fully dependent on those savings and financial support from the family. Other participants sold equipment or were able to find different employment options. One way to generate income was through private groups providing small loan options to members. However, in order to stay a member of this investment group and be allowed to access the loans, the participants sold items from their homes.

P329 (M): [ … ] Maybe in a week you give eleven thousands and later on you receive something, maybe later when you need a loan, so those changed too because you stay for several weeks without contributing, you are sick and you can’t work so you have several weeks without giving, we usually have plans in these groups that we will do this and this with these contributions, now there are things like that which stopped, so after coming back home I saw that it is hard to pay like my fellows do. There is a bicycle here, let me sell it and return it back there so I can be equal with them.

##### Financial Burden Impacting Emotional Well-Being

The family and patients reported varying degrees of emotional burden on the entire family. Those wanting to provide medical treatments, but being unable to afford that for the patient, suffered a great deal from financial hardship. The most commonly mentioned concern that affected the patient’s and family’s mental well-being was the inability to provide for the children and cover school-related costs. Families voiced hopelessness, feelings of frustration, fear, loneliness, and uncertainty about the future due to their financial situation. One participant reported the inability to sleep due to the pressure to provide for the family. Another emotional burden was the awareness that the lack of money was delaying or prohibiting them from receiving necessary care. The length of recovery was the source of lacking motivation for one patient, who self-reported mild but noticeable mental health effects due to the increased financial burden. One caregiver voiced the challenge of continuous substance use of the patient, which further strained the financial situation of the family.

INT: Ok, how do you think these economic barriers have affected your mental health?

P67 (M): Aah mental health, not much as I said. You are thinking about things but sometimes you just say these are normal things they will pass, I can’t say there are no effects, it affected me a little bit but not so much, because if the effects are big then they may bring up other things in the head, so it affected me a little bit.

#### 3.4.2. Access to Healthcare, Ability to Follow Healthcare Recommendations

##### Discharge Planning

Discharge planning and the process of transitioning back into their home setting was not commonly discussed by the participants. It was generally believed that only doctors have the authority and competence to provide information and recommendations on the patient’s health status and recovery process.

##### Injury Impacted Transportation Abilities

In the majority of cases, injury impacted transportation abilities, usually coinciding with increased costs. For example, participants mentioned that paying a taxi driver is more expensive than a bajaji and resting a leg requires two seats on a bajaji. In many cases, a caregiver had to be present for transportation.

##### Lack of Infrastructure Impacting Management of Care after Discharge

The patients were responsible for managing their care and overcoming logistical barriers, due to a lack of file transfer, communication, connectedness, and collaboration between facilities and services. The need to overcome those barriers also led to delays in care.

##### Limited Healthcare Provider/Resources Availability

Challenges in accessing continuous healthcare after discharge included difficulties in finding appropriate providers for follow-up care and having limited access to timely services and equipment. The need for referrals to access trained or specialized healthcare contributed further to access issues.

##### Compliance with Doctors’ Prescriptions

The patients and caregivers overall trusted the doctors’ recommendations and followed their orders as prescribed. This was reflected in both injury-related and general health behavior. Information provided by medical professionals included when and where to return for follow-up, additional medical steps (such as vaccination boosters), diagnostic imaging, and prescription of assistive devices and medical equipment. Patients felt education by doctors was necessary for them to understand their conditions and manage their expectations for recovery.

##### Pain Management Strategies

Pain management strategies were inconsistent and included medications, massages from the caregivers and other alternative strategies, and no strategy at all. Some reported pain medications to be ineffective. Others found it to be intolerable due to side effects. Concerns about becoming addicted to pain medication caused some participants to wait until their pain was intolerable to take the medication. Generally, information on addiction and side effects was not provided by healthcare providers upon discharge. One patient reported receiving information on the potential for addiction from a community member.

INT: Due to pain, you said you’re taking painkillers like tramadol, and other substances; by any chance are you addicted to any of those?

P240 (M): By good luck I got some information from my neighbor, the owner of [ … ] Pharmacy about drug use and painkillers like tramadol which, she said, can be addictive.

INT: Okay.

P240 (M): After I have been discharged with tramadol at KCMC, I wanted to use them, but she warned me that they might be addictive, so I should be careful. So, I had them just for emergencies; and I only took them when I could not tolerate any more pain.

##### Communication with the Healthcare Team

The importance of continuous communication with the physicians between follow-up visits was mentioned as an important component of the patients’ recovery. Patients voiced the need to receive better information on how they themselves could actively contribute to their recovery. During the hospital stay, dissatisfaction was voiced regarding the education the patients received and the lack of information sharing between the provider team.

##### Knowledge of How to Access Healthcare Resources after Discharge

The patients and caregivers had a generally good understanding of where to seek care, yet many did not specifically mention community resources as an access point after discharge. Many participants mentioned dispensaries and pharmacies or laboratories as their first point of contact in case of illness. In terms of awareness of healthcare-related costs, some participants mentioned they had health insurance to cover the costs of treatments, whereas others were unaware of the magnitude of healthcare-associated costs. Payments were due at every visit, which led to delays in care if the patients first had to borrow money.

INT: Ok, where or to whom do you usually go when you need health care?

P329 (M): For advice maybe? There is one woman, a neighbor who is a nurse, a close neighbor if there is something you may ask her, that there is this and this and she tells you just go to the hospital, do this, she is a nurse here at KCMC.

## 4. Discussion

The transition from hospital to home is a particularly vulnerable time for many patients. The home environment is less structured and supportive, medical bills accrue, new disabilities may affect patients’ ability to contribute as before, and families become responsible for coordinating care. Limited literature exists on this transitional period among trauma patients, especially in LMICs, such as Tanzania. Through interviews exploring the experiences of patients and their caregivers in Northern Tanzania after traumatic injury, seven major themes arose. These themes included pain, decreased physical functioning, emotional distress, lack of support, difficulties with daily activities, financial concerns, and obstacles to accessing healthcare. Applying the biopsychosocial model to our findings helped ensure a holistic approach to characterizing the transition of care challenges in the Tanzanian injury population.

### 4.1. Biological Factors

Pain was commonly introduced into conversation by patients and caregivers as a significant component of their transition home. Many reported that pain considerably impaired their functioning. Many patients relied on analgesic medication for pain control and uncertainty regarding prescribed medications as well as the general recovery timeline was common. Feeling uninformed at hospital discharge was one of the four themes that likewise emerged in an Australian transitions of care study [27]. In a study looking at transitions of care from hospitals to skilled nursing facilities, stakeholders also reported a high prevalence of communication gaps in expectations regarding plans for pain medication titration after transfer from the hospital setting [28]. Some patients in our study expressed concerns about the potential for addiction. However, one Canadian group reported that after major elective surgery, only 3.1% of patients who were previously opioid natives were still receiving opioid medications after 90 days [29].

Improving discharge practices, and thus medication literacy seemingly would address many of the problems expressed regarding experiences with pain. Expectation setting regarding the expected level of pain, recovery timeline, medication use guidelines, and the low risk of long-term opioid reliance would likely improve the ability of patients and caregivers to manage pain effectively after discharge.

### 4.2. Psychological Factors

During the transition of care period, many participants noted changes in their emotional health, citing the significant impact of the injury on daily life, financial issues, and uncertainty about the path to recovery as causes.

Psychological afflictions in the aftermath of physical trauma are common and seen in up to half of the patients one to two months after discharge [30]. Such problems are known to be associated with impaired functional status [31]. Specifically, post-injury depression and post-traumatic stress disorder have been shown to be independent predictors of poor scores on indices of functioning six months after injury [31].

Despite the prevalence and known harmful effects of emotional issues post-hospitalization for traumatic injury, few patients receive care. In one study, only 10% were obtaining professional help [30]. Not knowing how to obtain or where to go for help was a barrier cited by 42% of patients, and 79% responded “no” or “I don’t know” when asked if they had been provided information about managing emotional issues after discharge [30]. Financial barriers to such services are common, with 58% of participants in one study citing cost as a barrier to accessing mental health care [30]. The lack of affordability of health services, in general, caused our participants significant emotional distress.

Before discharge from the hospital, trauma patients would likely benefit from being set up with counseling and peer support resources. One solution successfully implemented in an American trauma center is the development of partnerships with mental health staff before discharge to help confront, predict, and plan the response to any emotional issues that may arise upon the patient’s return home [30]. Another possible approach is to connect families with patient and caregiver support initiatives [30].

Changes are also in order to decrease the toll financial hardships have on psychological well-being. Solutions include increased transparency about costs, income assistance programs, and policies protecting patients from financial hardship after injury. Changes to health insurance, such as ensuring that patients are covered by insurance plans that cover rehabilitation services, would also be beneficial [32].

### 4.3. Social Factors

An additional major theme from our discussions with patients and caregivers was that, during the transition period, community support was difficult to come by. Instead, the family was often cited as the main source of help and patients were generally unaware of existing supplementary avenues for social support.

Caregivers play an exceptionally important role in supporting patients in the aftermath of hospital discharge [33]. Previous work has been conducted looking into the nature of the caregiving relationship. One study in Peru analyzing the results of the Disability National Survey identified family members as caregivers in over 94% of dependents [34]. As a result of their newfound responsibilities, caregivers are often forced to significantly change their daily routine [34]. Although some caregivers report the work to be rewarding, others report it to be burdensome [35].

Inadequate education of family caregivers during the transition of care period is known to contribute to gaps in care [33]. Considering that the nature of caregiving is usually informal and can require adaptation and sacrifice on behalf of the caregiver, there is a need for caregiver support and training [34]. A Professional-Patient-Partnership model was used successfully in Baltimore, MD, USA, to assist caregivers of adults with heart failure [33]. Nurses and social workers received training on how to involve patients and caregivers in the discharge process, including assessing needs and providing information on discharge care and how to access community services [33]. As a result, patients felt more prepared for discharge and caregivers reported more role satisfaction after two weeks [33].

### 4.4. Limitations

There are multiple limitations in this study. Several involve the characteristics of patients and interviews. First, while the participant demographics may be representative of the injury population, they are not necessarily representative of the population as a whole. For instance, the study sample included injury patients living in or close to Moshi, thereby excluding participants in more rural areas, who may face different challenges than those in more urban settings closer to a tertiary hospital. Further, no participants required ICU care, an experience that may have posed different challenges after discharge. Additionally, we only collected data from a single hospital, which limits the generalizability of our findings. Moreover, we followed a convenience sampling strategy, which allowed for the recruitment of participants in a timely manner. However, convenience sampling has the risk of over- or under-representing the population, also limiting generalizability. We did our best to mitigate that risk by recruiting a large enough sample to be representative of the injury population presenting to KCMC.

An additional limitation is that interviews were conducted at only one time period. The recency of memories and lack of longitudinal information may have skewed our data and interpretations. Moreover, nearly 70% of interviews were conducted within 1–2 months of injury and the longest period of time between injury and interview was seven months (Table 1). Considering that all interviews were conducted relatively shortly after the patients’ injuries, we may be failing to represent any new challenges that arose as patients moved further from hospital discharge.

The revision made to the codebook during the data collection process is another limitation; however, the interview guide did not undergo substantial changes and interview procedures were not modified during the revision process. Further, although our research team made their best efforts to transcribe all interviews and focus group discussions ad verbatim because we did not return the transcripts to participants for correction, there may be instances of unintentional inaccurate reporting of participant statements. The potential for inaccuracy may have been enhanced by the subsequent Swahili-to-English translation process.

## 5. Conclusions

To our knowledge, this is the first study evaluating stakeholder experiences during the transition of care period after traumatic injury in LMICs. Pain, emotional afflictions, lack of avenues for support, and financial strain were commonly expressed concerns. We found the biopsychosocial model to be helpful for understanding the patient and caregiver experience. Advances in discharge practices, community support, and patient and caregiver education are necessary to improve the transition of care process in this study setting. Utilizing qualitative components and functional frameworks that allow a holistic view of the patient and the community will be critical in informing how the existing transition of care models from HICs can be adapted and implemented in LMICs.

## Figures and Tables

**Table 1.** Characteristics of Patients, Caregivers, and Injury.

**Patient Age, Mean (SD)**	37.7 (11.1)
**Male, N (%)**	21 (80.8)
**Residence, N (%)**	
Moshi Urban	15 (57.7)
Moshi Rural	10 (38.5)
Other	1 (3.8)
**Tribe, N (%)**	
Chagaa	16 (61.5)
Pare	2 (7.7)
Zigua	2 (7.7)
Other	6 (23.1)
**Marital Status, N (%)**	
Married	16 (61.5)
Single	9 (34.6)
Separated	1 (3.8)
**Years of Formal Education, N (%)**	
7	16 (61.5)
8–11	2 (7.7)
12–13	3 (11.5)
14–16	5 (19.2)
**Payment Method for Medical Costs, N (%)**	
Cash personal payment	18 (69.2)
National Health Insurance	7 (26.9)
Other	1 (3.8)
**Cause of Injury, N (%)**	
Road Traffic Injury	17 (65.4)
Assault	2 (7.7)
Fall	3 (11.5)
Other	4 (15.4)
**ICU stay, N (%)**	
Yes	0 (0.0)
No	25 (96.2)
Unknown	1 (3.8)
**Surgery performed, N (%)**	
Yes	18 (69.2)
**Time of interview since injury, N (%)**	
1–2 months	18 (69.2)
3–4 months	6 (23.1)
5–7 months	2 (7.7)
**Caregiver characteristics, N (%)**	
Wife	5 (45.4)
Sibling	2 (18.2)
Other (child, cousin, sister-in-law, grandmother)	4 (36.4)

**Table 2.** Overview of Major Themes and Sub-Themes.

Major Theme	Sub-Themes
**Biological Factors**	
Experiences and impact of pain	-
Perceived impact of injury on health and physical function	-
**Psychological Factors**	
Emotional and psychological well-being	Uncertainty about future; Impact on family members’ health; Impact on patients’ mental health; Unadjusted coping/failure to ask for support
**Social Factors**	
Community, friends, and family support	Level of community support/lack of support at home post-injury from community members; Injury leads to lack of active community engagement; Level of support from religious community; Level of family support; Injury impacted family daily activities; Expectations for the support system; Structured/organized support systems
Impact on activities of daily living	Injury impacting daily activities or ability to do housework; Growing ability to perform daily activities with less support; Environmental changes (house, assistive devices, transportation, etc.)
**Contextual Factors**	
Finances and income	Injury negatively impacted the ability to generate family income; Injury-related costs increase the financial burden on the family; Found new ways to generate income/savings to help cover costs; Financial burden impacting emotional well-being
Access to healthcare, ability to follow healthcare recommendations	Discharge planning; Injury impacted transportation abilities; Lack of infrastructure impacting management of care after discharge; Limited healthcare provider/resources availability; Compliance with doctors’ prescriptions; Pain management strategies; Communication with the healthcare team; Knowledge of how to access healthcare resources after discharge

## Data Availability

The data that support the findings of this study are available from the corresponding author, C.A.S., upon reasonable request.
